# Evaluation of Diagnosis Techniques Used for Spinal Injury Related Back Pain

**DOI:** 10.1155/2011/478798

**Published:** 2011-06-13

**Authors:** Meaghan Janssen, Aliaa Nabih, Walied Moussa, Gregory N. Kawchuk, Jason P. Carey

**Affiliations:** ^1^Department of Mechanical Engineering, University of Alberta, Edmonton, AB, Canada T6G 2G8; ^2^Faculty of Rehabilitation Medicine, University of Alberta, Edmonton, AB, Canada T6G 2G4

## Abstract

Back pain is a prevalent condition affecting much of the population at one time or the other. Complications, including neurological ones, can result from missed or mismanaged spinal abnormalities. These complications often result in serious patient injury and require more medical treatment. Correct diagnosis enables more effective, often less costly treatment methods. Current diagnosis technologies focus on spinal alterations. Only approximately 10% of back pain is diagnosable, with current diagnostic technologies. The objective of this paper is to investigate and evaluate based on specific criteria current diagnosis technique. Nine diagnostic techniques were found in the literature, namely, discography, myelography, single photon emission computer tomography (SPECT), computer tomography (CT), combined CT & SPECT, magnetic resonance imaging (MRI), upright and kinematic MRI, plain radiography and cineradiography. Upon review of the techniques, it is suggested that improvements can be made to all the existing techniques for diagnosing back pain. This review will aid health service developers to focus on insufficient areas, which will help to improve existing technologies or even develop alternative ones.

## 1. Introduction

Back pain is a prevalent condition affecting approximately 80% of the population at one time or another. Current technologies focus on diagnosing spinal alterations. Only approximately 10% of back pain is diagnosable, with current diagnostic technologies. Diagnosis is the first step to determining and starting treatment.

Nine diagnostic techniques were found through reviewing scientific literature and interviewing medical personnel, each which fulfills individual requirements to diagnose spinal alterations. The techniques include discography, myelography, single-photon emission computer tomography (SPECT), computer tomography (CT), combined CT and SPECT, magnetic resonance imaging (MRI), upright and kinematic MRI, plain radiography, and cineradiography. These techniques have been investigated and compared on defined criterion. This will provide insight into currently sufficient and insufficient areas within each technique.

## 2. Methodology

### 2.1. Determining Techniques

There are many techniques currently available to assess spinal injuries and back pain. Scholarly articles, medical textbooks, and medical personnel were used to compile a list of the techniques currently used. Compendex, PubMed, Web of Science, and the University of Alberta catalog were used to search diagnostic techniques. Initial searches involved combinations keywords. The keyword combinations and sequence can be seen in Table 1.

### 2.2. Classification

After information was collected from the databases, Medical Algorithms, Dynamed, the Cochrane library, HAPI, Embase, and Scopus were searched for further information. These databases only returned previously collected information. An assessment of the techniques began at this point. Manual indentation was not included in the assessed techniques because it is always used but does not provide specific information on the injury. 

**Figure 1:**
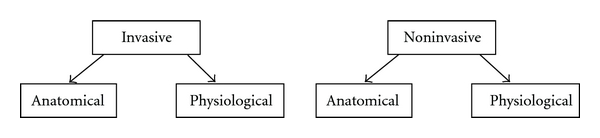
Method separation.

To provide an overview, the diagnostic techniques were first classified as either invasive or noninvasive. Technologies were further separated as anatomical or physiological as seen in Figure 1. 

### 2.3. Assessment Parameters

Assessment parameters to rate the techniques were developed. These parameters were selected in order to encompass patient, practitioner, and community concerns. The parameters used include time, cost, patient pain/discomfort, accessibility, accuracy/results, risks, and restrictions. 

#### 2.3.1. Time

The time parameter illustrates preparation, test, recovery, and interpretation time associated with the effectiveness of each technology.

#### 2.3.2. Cost

The cost parameter encompasses multiple factors including infrastructure, training, and operating costs. Infrastructure costs represent the equipment costs. Training costs represent the expenses of training personnel (technologists/physicians/nurses) in order to perform the exam and interpret the results. Operating costs represent the expenses associated with the usage of the techniques (i.e., staff, electricity, etc.).

#### 2.3.3. Patient Pain/Discomfort

Patient pain/discomfort represents the physical pain or amount of physical or mental discomfort a patient will experience during and following the procedure.

#### 2.3.4. Accessibility

Accessibility represents how readily available the procedures are for citizens of different population areas. Everything from large hospitals in major urban areas to general physician offices in rural areas was considered.

#### 2.3.5. Accuracy/Results

The accuracy/results specification describes the technologies' abilities to produce valuable results when diagnosing back pain. The techniques were evaluated based on their ability to distinguish location and nature of specific alterations.

#### 2.3.6. Risks

The risk consideration provides insight into the potential dangers that patients face. These dangers include those of the actual procedure as well as future risks.

#### 2.3.7. Restrictions

The restrictions parameter helps quantify when each technique would be able to be used. This illustrates the limitations of each technique. Weight restrictions of operating tables are common to all techniques.

### 2.4. Rating Techniques

Following the definition of parameters, the techniques were rated. A design matrix was created to illustrate the techniques ratings. Techniques were rated as good, fair, or poor relative to each other. The design matrix was not weighted, as weighting would depend on the stakeholder. All monetary values are given in Canadian dollars.

## 3. Results

### 3.1. Techniques

The nine diagnostic technologies that were found through the literature review are classified in Table 2. Following is a description of each technique.

#### 3.1.1. Discography

Discography assesses which disk is causing a patient pain. A radio-opaque dye is injected into the suspected disk(s) followed by an X-ray which examines the disk anatomy and any dye leaks [1–3]. This procedure may be followed by a CT scan, which is able to further detect internal disk disruptions.

#### 3.1.2. Myelography

Myelography involves injection of a contrast material into the spinal column and records the motion of the contrast material with fluoroscopic imaging. The dye will be blocked or diverted if an abnormality is present [4, 5]. This procedure may be followed by a CT scan, which improves the diagnostic accuracy of the test.

#### 3.1.3. SPECT

SPECT is a nuclear medicine technique used for bone scans of the spine. After the injection of a radioactive tracer, technetium-99m, gamma cameras detect the energy emitted by the tracer and use this information to create pictures which record the intensity and distribution of the tracer in the tissue [6–11]. The images provide an internal view of the body. This enables precise determination of a lesion [9, 12, 13].

#### 3.1.4. CT Scan

CT scans use X-rays to produce multiple, cross-sectional images of the body. The images produce an internal view of the body. CT scans enable the structure of the vertebrae and the intervertebral disks to be accurately displayed. CT scans display the spine in multiple planes, with a three-dimensional imaging option available, on a computer screen [14].

#### 3.1.5. SPECT and CT

Historically, functional and anatomical imaging techniques was done independently. Correlation of images from the different imaging techniques were done by side-by-side comparison [15]. Hardware fusion of certain techniques can overcome the inaccuracies resulting from this comparison, enabling software fusion of images [15–17]. 

The diagnostic accuracy of improved localization and definition of specific lesions, resulting from the hardware fusion of SPECT with CT, is well known [15–17]. There is a software system being developed to validate the anatomical accuracy of this system [18]. A range of SPECT/CT scanners exist, commencing a from a low radiation dose four slice CT scanner being added on to a SPECT machine to a fully integrated SPECT and multidetector CT system [15, 19]. Essentially one test is performed directly after the other and the images are overlaid.

A range of SPECT/CT scanners exist, commencing a from a low-radiation dose 4-slice CT scanner being added on to a SPECT machine to a fully integrated SPECT and multidetector CT system [15, 19]. A patient receives both internal and external radiation from this system. The external dosage of radiation varies according to the CT component of the system. The internal component of radiations is quite consistent at 5.7 *μ*Sv [17]. 

#### 3.1.6. Plain Radiography

The plain radiography procedure consists of sending ionizing radiation (X-rays) through the body, which produces a one-dimensional exposed film or digital image. The different components of a body absorb different amounts of these X-rays [20, 21]. The X-ray images are seen as looking through the body. The radiation dose is approximately 20 *μ*Sv [22].

#### 3.1.7. MRI

MRI uses a powerful magnetic field, radio wave pulses, and a computer to generate accurate, detailed pictures of all internal body structures. MRI presents a view to physicians as though they had opened up the body. Spine MRIs allow the vertebrae and intervertebral disks as well as the spinal cord and other tissues in this area to be investigated [23].

#### 3.1.8. Cineradiography

Cineradiography uses fluoroscopic X-rays to record a film during patient motion. This video shows abnormalities in the spine and specifically abnormalities with spinal motion [24]. Cineradiography is used to evaluate lumbar motion in flexion and extension and to quantify this data to present normal and abnormal motion [25–30].

#### 3.1.9. Upright and Kinematic MRI

Upright and kinematic MRIs image a patient in a position other than the traditional recumbent position of MRI. Standing position takes into account gravitational and weight-bearing effects. A patient may be imaged in the specific position which causes pain. Though there is much information on the validity of this type of imaging [31–35], there is much contradictory information on what this technology accurately diagnoses and what it is most useful for [36]. This technology is relatively new and is currently being further investigated.

### 3.2. Evaluation of Techniques

Following is an evaluation of each technique according to the established assessment parameters. The technique description chronologically responds to stated assessment parameters of time, cost, patient pain/discomfort, accessibility, accuracy/results, risks, and restrictions. 

#### 3.2.1. Discography

Discography is no longer commonly used [37]. The procedure takes approximately 30–45 minutes to complete, and patients are advised to have someone else drive them home and to relax for a couple days following the procedure [3, 38]. A significant amount of information is obtained during the procedure from the patient pain response [2]. The attending physician will then interpret the results.

This procedure is performed in designated special room and requires fluoroscopy equipment costing approximately $500,000 [39]. The dye injection requires a physician, and a technician will be present to operate the fluoroscopy equipment. Alberta health pays approximately $200 for this procedure [40].

Patients may experience varying degrees of pain when the radio-opaque dye is injected into the suspected intervertebral disc(s). The patient may experience no pain, pain similar to their regular symptom, or a different pain entirely. The pain the patient experiences is useful in determining which (if any) intervertebral disc(s) is causing the patients pain [38]. Patients are required to lie on their stomach during this procedure, and this position may be uncomfortable for certain individuals.

Discography is only available in certain large hospitals that still have the equipment and staff to perform it [37, 41].

Discography is the only available procedure which can determine precisely which disk is causing pain. Discography is able to determine if an abnormal suspect disk is the source of the patients pain or not [2]. 

The disruption of a nonpainful or painful intervertebral disk carries serious risks with it. Intervertebral disks are self-contained pressure zones, and disrupting this zone may affect the integrity of the disk and risks increasing patient pain. There is much controversy as to the use of this technique [1, 2, 42, 43]. Discography also carries the regular risks associated with invasive test, such as infection bleeding and. There is a slight risk thatthe needle will brush a nerve when it is inserted and cause persisting pain for a few days [38].

Discography is provided as a last result to diagnose abnormal disks. Discography is only offered to patients who have already tried medications or activity recommendations to treat their problems [38]. Discography will not be performed if the patient is allergic to the injected dye or if a patient is on blood thinning medications.

#### 3.2.2. Myelography

Myelography is no longer commonly used. Myelography is normally used if a patient has an internal medical device which restricts the use of an MRI machine [5, 44]. The test takes approximately 30–60 minutes. This exam length is followed by a period of observed recovery which can last up to 4 hours. At such time the patient will be discharged and recommend to keep their head elevated above their body for 8 hours to provide time for the dye to absorb. Patients are also recommended to relax for a couple days following the procedure [4, 5]. Myelography results are sent to a physician for interpretation.

This procedure is performed in a special room and requires fluoroscopy equipment costing approximately $500,000 [39]. A physician is required to inject the contrast material into the spinal canal, and a technician is required to run the fluoroscopic X-ray equipment. Alberta health pays approximately $150 for this procedure [45].

Patients may experience varying degrees of discomfort/pain when the contrast material is injected into the spinal canal. Prior to contrast injection, the injection site is numbed with a small needle. The contrast may cause patients to experience nausea, headaches, or more severe symptoms during or following the procedure [4, 5]. Patients are required to lie still on their stomach or side for the duration of the test which may be quite uncomfortable. 

Myelography is currently only available at large urban hospitals [41]. This procedure has been all but replaced by MRIs and CT scans. Myelography is commonly only performed when a patient has a factor which restricts them from receiving an MRI or CT scan, such as metal implants, claustrophobia, or device weight restrictions. 

The path of the contrast material is tracked through the patient's spinal canal which will indicate where abnormalities exist. Myelography does not provide any specific identification of the abnormalities unless they are visibly determinable from the contrast path. Patient movement may obscure results [42, 43, 46–48].

There are significant risks associated with the injection of contrast into the spinal canal [46–48]. A patient must, during the procedure and subsequently for 8 hours, keep their head elevated above their body to prevent dye from leaking into their head. If the dye does leaks into one's head it has results varying form a headache and nausea to a seizure. The injection site of the needle poses a risk that it may not close properly, potentially allowing spinal fluid to drain out, resulting in the requirement of further procedures to stop the leak. In some cases extremely serious effects such as paralysis or loss of bowel/bladder control may result [4, 5]. 

Myelography is restricted by patients allergies to the contrast agent used. The major restriction, of the myelography procedure, is that myelography only allows the physician to see abnormalities within the spinal canal and nerves roots surrounding this. Myelography does not record with any certainty, abnormalities outside of the spinal canal. This procedure is often avoided for pregnant women due to the risk to the fetus. Myelography may not be an option for patient with structural spinal issues or following certain spinal surgeries due to difficulties in properly injecting the contrast material [4, 5].

#### 3.2.3. SPECT

SPECT technology is no longer commonly used as the hybrid SPECT and CT scanners are now widely implemented. A SPECT scan requires a patient to receive isotope injection and then wait three hours for the isotopes to distribute. After this waiting period the actual exam takes approximately 35 minutes [8]. After the procedure the patient is free to leave, and the radioactive isotope should be completely eliminated from ones system via urine within 24 hours [49]. SPECT results are sent to a physician for interpretation.

This procedure requires gamma camera with an approximate cost of $850,000 [39].This procedure is performed by a nurse and a technologist. The nurse is able to inject the radioactive tracer intravenously into a patient, and the technician operates the gamma camera. The operational cost encompasses the injection of the technetium-99m and the imaging procedure. Alberta health pays $387.97 for a bone scan [50].

The SPECT procedure is only minimally invasive with the radioactive tracer being injected intravenously [7, 49]. This tracer does not normally cause patient discomfort. The patient is required to remain still or move according to the technologist's direction during the procedure; these requirements may be uncomfortable for patients. 

The first use of SPECT for spinal imaging was published in 1984 [13]. However, this technique has not become common practice. This technique is currently used at the large hospitals [8, 41]. 

This technology is able to provide the precise location of a lesion in the vertebral body, arc, or disk space [9, 12, 13]. The SPECT detectors can be obliquely orientated which enables them to be positioned parallel to the anatomical plains and provide a clear view of the anatomical structure [9, 12]. This technique is also able to distinguish between recent and old injuries due to the tracer intensity [7, 51].

The radiation exposure due to a SPECT scan is due to the radioactive tracer. This tracer causes internal radiation to a person which is approximately equivalent to the external radiation dose received through a chest X-ray [49, 52]. The actual effects of internal radiation are poorly understood. The radioactive tracer also carries risks of vomiting and dizziness. 

This procedure is normally avoided for pregnant women due to the unknown risk to the fetus. Breastfeeding women are also recommended not to nurse the children for 36 hours following the procedure [52].

#### 3.2.4. CT Scan

Through new developments CT scans are becoming increasingly patient friendly. CT scanners are now able to achieve information from a chest exam that previously required 10 separate breath holds of 10 seconds in each one of these breath holds. CT scanners are now able to reconstruct a study of 1,000 images in less than 30 seconds [22]. A CT scan for one spinal section takes between 15–30 minutes to complete [14, 53]. Patients do not require any rest or recovery period prior or following the exam. A radiologist is required to interpret the results.

A CT machine costs approximately $1,000,000 [39]. This procedure is performed in a special room to limit radiation exposure. A technician performs this procedure. This exam costs approximately $360 per spinal section [54, 55].

The CT procedure does not hurt the patient. However, being required to remain still for the procedural time may be uncomfortable for some patients. CT scans allow some room for slight patient movement before the results will be obscured [14, 56]. Some patients may experience increased anxiety due to the spatial constraints of CT scanners.

CT scans are available in main hospitals in main urban centers and at private clinics [41]. 

This technique provides multiple, cross-sectional images of the body [14, 56, 57]. CT scans enable the structure of the vertebrae and the intervertebral disks to be accurately shown. 

CT scans expose the patient to large radiation doses. One whole body CT scan results in a radiation dose of 4 to 24 mSv to the patient. This radiation dose varies by a factor of ten dependant on the size of the individual, the equipment used, operating techniques, and procedures followed. To put this radiation dose in perspective, a radiation dose of 10 mSv, the equivalent of 500 chest X-rays, results in a risk of cancer death of 1 in 2000 [22]. The potential benefit of correctly diagnosing a spinal fracture being more significant than this risk is a controversial topic [22, 56, 58]. The separation of patient from medical devices, during the performance of a CT scans in emergency situation, posses a potential risk to the patient [14].

Pregnant women are advised strongly against having a CT scan. The closed confinement setup of a CT scanner restricts the size of patients and is restricted to claustrophobic patients. Similar to plain radiographs, CT scans only provide valuable information about the bone, not ligaments and intervertebral disks [14].

#### 3.2.5. SPECT and CT

SPECT and CT requires the same procedures for SPECT to be performed injection, wait time, and procedural time, plus additional time for the CT scan. The CT scan takes approximately 20 minutes [59]. A radiologist is required to interpret the results.

A SPECT and CT scan system costs approximately $1,400,000 [39]. This procedure is performed in a special room where a nurse performs the intravenous injection of radiotracer and a technician operates the machine. 

All the discomforts associated with both SPECT and CT scanning alone are present. 

This technique is available at hospitals in large urban areas [41]. This technique was implemented by large Alberta hospital in 2007 [59]. 

The hardware fusion technique provides better comparison of results and improves upon the accuracy of either technique alone [15–18]. 

Risks associated with both CT scans and SPECT are present. A patient receives both internal and external radiation. 

This technique encompasses all the restrictions of each individual technique. This fusion overcomes CT restrictions of only clearly demonstrating the bone.

#### 3.2.6. Plain Radiography

The plain radiography test takes approximately 15 minutes to perform, and patients do not require any rest or recovery period [60]. The efficiency of plain radiography is continually improving, with radiographers being increasingly able to report in accident and emergency settings (12.2% increase in four years). Radiographer's potential to report in clinical settings is being investigated. Radiographers have been shown to have 92.6% sensitivity and 97.7% specificity when compared with radiologist diagnosis [61, 62]. Due to the increase in radiographers reporting the interpretation time for plain radiography is continually decreasing.

Plain radiography equipment is widely available and costs approximately $500,000 [39, 41]. This procedure is required to be performed in a specialized room to block radiation exposure to others. There are also portable forms of plain radiography machines available. This procedure may be performed by a technician alone. Alberta health pays $61.62 per spinal section radiograph [63].

Patients are required to remain still during the procedure and may be placed in uncomfortable positions.

There are many radiology clinics. All hospitals have the equipment to take these X-rays [41]. 

Plain radiography enables physicians to diagnose large spinal abnormalities. However, smaller fractures or other abnormalities, including internal abnormalities, are not distinguishable through this procedure [64, 65]. Patient movement during the exam may obscure the results. In Canada there are established screening guidelines for C-spine radiography use in trauma patients, the Canadian C-spine (cervical spine) Rule (CCR) and the National Emergency X-Radiography Utilization Low-Risk Criteria (NLC).Though these guidelines exist it still falls to the attending physician to determine what X-rays should be taken. As well there are no established guidelines for screening the thoracolumbar spine section [66, 67], which results in thoracolumbar spinal injuries being potentially overlooked in patients with multiple injuries or who are drowsy or intoxicated [64, 65]. A recent study has shown that 8.2% of patients with traumatic injuries to the lumbar spine, due to a blunt trauma or high-energy impact situation, would have been overlooked for a CT scan, based on plain radiography findings, which would have demonstrated this injury [58].

Plain radiography exposes the patient to minimal ionizing radiation. For a spinal the radiation dose received is equivalent to a few months to year of background radiation exposure. This radiation dose is approximately 0.3 mSv [21, 68].

Pregnant women are advised against having plain radiography performed. Plain radiographies only provide information about the bone, not ligaments and intervertebral disks [20, 60].

#### 3.2.7. MRI

An MRI exam takes approximately 30–45 minutes to perform [53]. Patients do not require any rest or recovery period. The results are sent to a radiologist for interpretation.

The MRI machine costs approximately $2,000,000 [39]. This procedure is performed in its own room to isolate the effects of the equipment and the noise. A technologist performs this procedure. An MR imaging scan of one spinal section costs approximately $660 [69, 70].

Patients are required to remain still for the duration of this procedure [23]. Spatial constraints may cause patient anxiety. Patients are faced with a loud unpleasant noise produced by the operating equipment.

MRI equipment is available at main hospitals in large urban centers, also in private clinics. MR imaging is slightly less available than CT scans.

Spine MRIs allow the vertebrae and intervertebral disks as well as the spinal cord and other tissues in this area to be investigated [71]. This method of testing enables detections of abnormalities that may have been obscured by bones in other tests. MR imaging is able to detect a bone bruise with no fracture present [23].

MRI, during emergency procedure, requires the patient to be separated from life-saving. MRI does not use ionizing radiation. The magnetic field generated causes no direct risk to patients. However, the field may cause the failure of implanted medical devices such as a pace maker [23].

The dimensions of an MRI machine restrict certain body types. This confinement may also affect claustrophobic patients. Patients with metal implants are restricted from receiving such an exam. Pregnant women are advised against having an MRI, although, there is no evidence proving a negative effect on a fetus [23].

#### 3.2.8. Cineradiography

Cineradiography is not widely used to evaluate the spine; though DFV is being developed to have a higher use in diagnosing spinal injuries [72]. The procedural time varies according to how many X-rays are to be taken. Patients do not require any rest or recovery period. Results are sent to a radiologist for interpretation.

The fluoroscopy equipment required costs approximately $500,000 [39]. A technician performs this procedure. Alberta health pays $61.97 for fluoroscopy of a joint [73].

Cineradiography requires the patient to move in order to record the spinal motion for future analysis [74]. This motion may cause discomfort.

Cineradiography is accessible in many hospitals [41].

Cineradiography provides the physician with a kinematic view of the spine. This may allow for the detection of abnormalities in the spine not visible with a stationary X-ray [24]. Cineradiography is used to evaluate lumbar motion in flexion and extension and to quantify this data to present normal and abnormal motion [25–30]. Contradictory results have been presented on deformation patterns shown during flexion [26–28]. This resulted in the enhancement of fluoroscopic video techniques. Objective spinal motion imaging assessments (OSMIAs) are attempting to attain reliable frame-to-frame registration and objective consistent results in patterns [25]. Also a new more reliable, faster, higher-resolution digital fluoroscopic video (DFV) has been developed and is waiting for cross-validation before being clinically implemented. This DFV and distortion-compensated roentgen analysis are able to discriminate between patients with lower back pain and those without [29, 30]. The results observed of this new DFV technique serve as an initial step in utilizing this technique for widespread diagnosis of spinal injuries [30].

Cineradiography utilizes ionizing radiation. The exposure to this radiation varies according to duration of the exam.

Pregnant women are advised against having this. This procedure provides a view of the interaction of spinal vertebrae but does not provide an internal view of these vertebrae, nor does it provide a view of intervertebral disks and ligaments [24, 72].

#### 3.2.9. Upright and Kinematic MRI

Upright and kinematic MRI exams take approximately 30–45 minutes. Patients do not require any rest or recovery period. Results are sent to a radiologist for interpretation.

Upright and kinematic machines cost approximately $3,000,000 [39]. These machines are operated by a technologist. 

These technologies tend to image a patient in the position that causes their pain [31, 32], therefore causing the patient pain. These techniques require the patient to stay still for a period of time in specific positions [33–36]. These imaging devices have the negative noise of a regular MRI.

These technologies are available at major clinics in large urban centers [41]. 

These types of imaging improve upon recumbent MR imaging's ability to detect spinal abnormalities. These imaging modalities enable the weight-bearing effects of the spine to be represented [32, 34–36]. Also abnormalities associated with specific positions can be determined. 

These procedures pose the same risks to patients as regular MRI machines.

These new technologies overcome spatial restrictions of traditional MRIs. Though there is no evidence to support negative effect on fetus, pregnant women are advised against having such an exam. These exams are limited in by patient's abilities as it requires a patient to remain in awkward positions for lengthy periods of time.

## 4. Discussion

The evaluation of specified diagnostic technologies enabled them to be compared using assessment parameters. The results are shown in Table 3.

Discography determines which intervertebral disc is causing the patient's pain. This technique is expensive, painful, risky, and not highly accessible. 

Myelography provides similar, but not as comprehensive, results as MRI. This technique is highly invasive [46–48], time consuming, costly, painful, risky, and not highly accessible.

SPECT provides a cross-sectional imaging comparable to CT and MRI. SPECT is also able to distinguish between old and new injuries. This technique requires significant time to perform and is only accessible in large urban areas.

CT scans are quick and are able to accurately diagnose vertebral abnormalities. CT scans carry high associated risks due to the radiation dose.

Combined SPECT and CT presents views of both the vertebra and soft tissues. This technique carries high radiation doses with high cost and time and is only accessible in large urban centers. 

Plain radiography is widely available, quick, and inexpensive. This technology exposes the patient to minimal X-ray radiation. However, the accuracy of this technology is not guaranteed, and the X-rays taken are dependent on physician preferences.

MRI provides a safe and accurate diagnostic method. This procedure is expensive.

The developments currently being made with cineradiography are enhancing its ability to diagnose spinal abnormalities. At its current state, cineradiography is quick and minimally uncomfortable.

Upright and kinematic MRIs improve upon the uses of traditional MRI machines by accounting for weight-bearing and posture effects. The decreased patient size restrictions and increased range of motion imaging raise the cost; these factors also lower the accessibility. 

It should be noted that contrast-enhanced MRI and CT methods, which have the same disadvantages as traditional MRI and CT scans, have been showed to allow for an improved assessment of the variations in tissues which could possibly be attributable to LBP. Contrasted MRI has been shown to be superior to contrasted CT postdiskectomy procedures [75, 76]. 

The assessment has presented important factors relating to the use of diagnostic imaging techniques. No single technique is suitable for every situation, and no single technique will be sufficient by itself in all cases. The goal of any health care system should be to diagnose and treat a problem as quickly as possible, and with the least associated cost. All assessed techniques possess strengths and have areas that could be improved.

## 5. Conclusion

Improvements could be made to all the existing techniques for diagnosing back pain. This paper has highlighted sufficient and insufficient areas for discography, myelography, single-photon emission computer tomography (SPECT), computer tomography (CT), combined CT and SPECT, magnetic resonance imaging (MRI), upright and kinematic MRI, plain radiography, and cineradiography. This paper will aid health service developers to focus on insufficient areas, which will help to improve existing technologies or even develop alternative ones. 

Complications, including neurological ones, can result from missed or mismanaged spinal abnormalities. These complications often result in serious patient injury and require more medical treatment. Correct diagnosis enables more effective, often less costly treatment methods.

## Figures and Tables

**Table 1:** Keyword sequence used in databases.

Level 1
Spine + “back pain” + “diagnostic imaging”
Spine + “lower back pain” “diagnosis imaging”
Spine + “medical imaging”

Level 2 (includes level 1)
Lumbar
Thoracic
“Spinal injury”

Level 3 (includes 1 and possibly 2)
Diagnostic technologies
X-rays
CT scan
MRI
Radiography
Advantages
Scanning
Limitations
Ultrasound

Level 4 (Includes 1 and possibly 2 and 3)
Missed
Evaluation
Nuclear imaging
Thoracolumbar
Nuclear medicine,
Digital motion X-ray
Fluoroscopy
Upright MRI
*t*-spine
Discography
Standing
Myelography

Level 5 (includes 1 and possibly 2, 3 and 4 )
Bone scan
Contrast
SPECT
Pet scan

**Table 2:** Technique classification.

Techniques	Principal		Secondary	
	Invasive	noninvasive	Anatomical	Physiological
Discography	*✓*		*✓*	
Myelography	*✓*			*✓*
SPECT	*✓*			*✓*
CT Scan		*✓*	*✓*	
SPECT and CT	*✓*		*✓*	*✓*
Plain Radiography		*✓*	*✓*	
MRI		*✓*	*✓*	
Cineradiography		*✓*		*✓*
Upright and Kinematic MRI		*✓*	*✓*	

**Table 3:** Assessment matrix.

	Discography	Myelography	SPECT	CT Scan	SPECT and CT	Plain Radiography	MRI	Cineradiography	Upright a Kinematic MRI
Time	Fair	Poor	Poor	Good	Poor	Good	Fair	Good	Fair
Cost	Poor	Poor	Fair	Fair	Poor	Good	Poor	Fair	Poor
Patient Pain/Discomfort	Poor	Poor	Fair	Fair	Fair	Good	Fair	Good	Fair
Accessibility	Poor	Poor	Poor	Fair	Poor	Good	Fair	Fair	Poor
Accuracy/Results	Good	Fair	Good	Good	Good	Poor	Good	Fair	Good
Risks	Poor	Poor	Fair	Poor	Poor	Fair	Good	Fair	Good
Restrictions	Fair	Fair	Fair	Fair	Fair	Fair	Fair	Fair	Fair

## References

[1] Saboeiro GR (2009). Lumbar discography. *Radiologic Clinics of North America*.

[2] Manchikanti L, Glaser SE, Wolfer L, Derby R, Cohen SP (2009). Systematic review of lumbar discography as a diagnostic test for chronic low back pain. *Pain Physician*.

[3] Discography. http://www.spinedallas.com/discography-injection-interventional-spine-pain-doctor-dallas-tx.html.

[4] http://www.webmd.com/back-pain/myelogram-16147.

[5] Myelography. http://www.radiologyinfo.org/en/info.cfm?pg=myelography.

[6] Bodner RJ, Heyman S, Drummond DS, Gregg JR (1988). The use of single photon emission computed tomography (SPECT) in the diagnosis of low-back pain in young patients. *Spine*.

[7] Croft A (1996). Radionuclide bone scan: how useful in cervical spine trauma?. *Dynamic Chiropractic*.

[9] Sarikaya I, Sarikaya A, Holder LE (2001). The role of single photon emission computed tomography in bone imaging. *Seminars in Nuclear Medicine*.

[10] Gamie S, El-Maghraby T (2008). The role of PET/CT in evaluation of facet and disc abnormalities in patients with low back pain using 18F-fluoride. *Nuclear Medicine Review*.

[11] Suetens P (2002). *Fundamentals of Medical Imaging*.

[12] Gates GF (1996). Oblique angle bone SPECT imaging of the lumbar spine, pelvis, and hips: an anatomic study. *Clinical Nuclear Medicine*.

[13] Starck SA, Ohlsson J, Carlsson S (2003). An evaluation of reconstruction techniques and scatter correction in bone SPECT of the spine. *Nuclear Medicine Communications*.

[14] CT of the spine. http://www.radiologyinfo.org/en/info.cfm?pg=spinect.

[15] O’Connor MK, Kemp BJ (2006). Single-photon emission computed tomography/computed tomography: basic instrumentation and innovations. *Seminars in Nuclear Medicine*.

[16] Nömayr A, Römer W, Strobel D, Bautz W, Kuwert T (2006). Anatomical accuracy of hybrid SPECT/spiral CT in the lower spine. *Nuclear Medicine Communications*.

[17] Townsend DW (2008). Multimodality imaging of structure and function. *Physics in Medicine and Biology*.

[18] Han J, Köstler H, Bennewitz C, Kuwert T, Hornegger J (2008). Computer-aided evaluation of anatomical accuracy of image fusion between X-ray CT and SPECT. *Computerized Medical Imaging and Graphics*.

[19] Hamann M, Aldridge M, Dickson J, Endozo R, Lozhkin K, Hutton BF (2008). Evaluation of a low-dose/slow-rotating SPECT-CT system. *Physics in Medicine and Biology*.

[20] Smirniotopoulos JG PLAIN RADIOGRAPHS. http://rad.usuhs.mil/rad/home/plainrad.html.

[22] Elliott A (2009). Issues in medical exposures. *Journal of Radiological Protection*.

[23] MRI of the Spine. http://www.radiologyinfo.org/en/info.cfm?pg=spinemr.

[24] Cineradiography of the spine or dynamic motion x-Ray.

[25] Breen AC, Muggleton JM, Mellor FE (2006). An objective spinal motion imaging assessment (OSMIA): reliability, accuracy and exposure data. *BMC Musculoskeletal Disorders*.

[26] Hino H, Abumi K, Kanayama M, Kaneda K (1999). Dynamic motion analysis of normal and unstable cervical spines using cineradiography: an in vivo study. *Spine*.

[27] Kanayama M, Tadano S, Kaneda K, Ukai T, Abumi K, Ito M (1995). A cineradiographic study on the lumbar disc deformation during flexion and extension of the trunk. *Clinical Biomechanics*.

[28] Takayanagi K, Takahashi K, Yamagata M, Moriya H, Kitahara H, Tamaki T (2001). Using cineradiography for continuous dynamic-motion analysis of the lumbar spine. *Spine*.

[29] Teyhen DS, Flynn TW, Bovik AC, Abraham LD (2005). A new technique for digital fluoroscopic video assessment of sagittal plane lumbar spine motion. *Spine*.

[30] Teyhen DS, Flynn TW, Childs JD (2007). Fluoroscopic video to identify aberrant lumbar motion. *Spine*.

[31] Alyas F, Connell D, Saifuddin A (2008). Upright positional MRI of the lumbar spine. *Clinical Radiology*.

[32] Gilbert JW, Wheeler GR, Lingreen RA (2008). Imaging in the position that causes pain. *Surgical Neurology*.

[33] Gilbert JW, Wheeler GR, Kreft MP (2008). Repeat upright positional magnetic resonance imaging for diagnosis of disorders underlying chronic noncancer lumbar pain. *Journal of Manipulative and Physiological Therapeutics*.

[34] Suzuki F, Fukami T, Tsuji A, Takagi K, Matsuda M (2008). Discrepancies of MRI findings between recumbent and upright positions in atlantoaxial lesion. Report of two cases. *European Spine Journal*.

[35] Morishita Y, Hymanson H, Miyazaki M (2008). Kinematic evaluation of the spine: a kinetic magnetic resonance imaging study. *Journal of Orthopaedic Surgery*.

[36] Gedroyc WM (2008). Upright positional MRI of the lumbar spine. *Clinical Radiology*.

[38] Discography (Discogram) FAQs. http://www.reddinganesthesia.com/discogram.htm.

[41] Alberta I Service directory. informalberta.ca.

[42] Jackson RP, Cain JE, Jacobs RR, Cooper BR, McManus GE (1989). The neuroradiographic diagnosis of lumbar herniated nucleus pulposus: I. A comparison of computed tomography (CT), myelography, CT-myelography, discography, and CT-discography. *Spine*.

[43] Jackson RP, Cain JE, Jacobs RR, Cooper BR, McManus GE (1989). The neuroradiographic diagnosis of lumbar herniated nucleus pulposus: II. A comparison of computed tomography (CT), myelography, CT-myelography, and magnetic resonance imaging. *Spine*.

[46] Thornbury JR, Fryback DG, Turski PA (1993). Disk-caused nerve compression in patients with acute low-back pain: diagnosis with MR, CT myelography, and plain CT. *Radiology*.

[47] Thornbury JR, Fryback DG, Turski PA (1993). Erratum: disk-caused nerve compression in patients with acute low-back pain: diagnosis with MR, CT myelography, and plain CT. *Radiology*.

[48] Wildermuth S, Zanetti M, Duewell S (1998). Lumbar spine: quantitative and qualitative assessment of positional (upright flexion and extension) MR imaging and myelography. *Radiology*.

[49] Bone Scan. http://www.mdguidelines.com/bone-scan.

[51] Gates GF (1998). SPECT bone scanning of the spine. *Seminars in Nuclear Medicine*.

[52] http://www.brighamandwomens.org/nuclearmedicine/patient/bone.aspx.

[53] http://spinephysicians.org/.

[54] Mayfair Diagnostics. http://www.mayfairdiagnostics.com/.

[55] Timelymedical alternatives. http://www.timelymedical.ca/.

[56] Laughlin S, Montanera W (1998). Central nervous system imaging. When is CT more appropriate than MRI?. *Postgraduate Medicine*.

[57] Engelke K, Mastmeyer A, Bousson V, Fuerst T, Laredo JD, Kalender WA (2009). Reanalysis precision of 3D quantitative computed tomography (QCT) of the spine. *Bone*.

[58] Deunk J, Brink M, Dekker HM (2009). Routine versus selective computed tomography of the abdomen, pelvis, and lumbar spine in blunt trauma: a prospective evaluation. *Journal of Trauma—Injury, Infection and Critical Care*.

[60] Bone X-ray. http://www.radiologyinfo.org/en/info.cfm?pg=bonerad.

[61] Brealey S, Scally A, Hahn S, Thomas N, Godfrey C, Coomarasamy A (2005). Accuracy of radiographer plain radiograph reporting in clinical practice: a meta-analysis. *Clinical Radiology*.

[62] Brealey S, Hewitt C, Scally A, Hahn S, Godfrey C, Thomas N (2009). Bivariate meta-analysis of sensitivity and specificity of radiographers’ plain radiograph reporting in clinical practice. *British Journal of Radiology*.

[64] King SW, Hosler BK, King MA, Eiselt EW (2002). Missed cervical spine fracture-dislocations: the importance of clinical and radiographic assessment. *Journal of Manipulative and Physiological Therapeutics*.

[65] Poonnoose PM, Ravichandran G, McClelland MR (2002). Missed and mismanaged injuries of the spinal cord. *Journal of Trauma—Injury, Infection and Critical Care*.

[66] Ehrlich PF, Wee C, Drongowski R, Rana AR (2009). Canadian C-spine rule and the national emergency X-radiography utilization low-risk criteria for C-spine radiography in young trauma patients. *Journal of Pediatric Surgery*.

[67] O’Connor E, Walsham J (2009). Review article: indications for thoracolumbar imaging in blunt trauma patients: a review of current literature. *Emergency Medicine Australasia*.

[68] Wall BF, Hart D (1997). Revised radiation doses for typical X-ray examinations: report on a recent review of doses to patients from medical X-ray examinations in the UK by NRPB. *British Journal of Radiology*.

[69] Timelymedical alternatives. http://www.timelymedical.ca/.

[70] Mayfair Diagnostics. http://www.mayfairdiagnostics.com/.

[71] Afzal S, Akbar S (2009). Magnetic resonance imaging of lumbar intervertebral discs in elderly patients with minor trauma. *European Journal of Radiology*.

[72] Digital Motion X-Ray. http://www.dmxworks.com/html/research.html.

[74] Harada M, Abumi K, Ito M, Kaneda K (2000). Cineradiographic motion analysis of normal lumbar spine during forward and backward flexion. *Spine*.

[75] Annertz M, Hägglund G, Holtås S, Jönsson B, Strömqvist B (1992). Contrast-enhanced MRI versus myelography and contrast-enhanced CT in postdiskectomy problems. *European Spine Journal*.

[76] Malfair D, Beall DP (2007). Imaging the degenerative diseases of the lumbar spine. *Magnetic Resonance Imaging Clinics of North America*.

